# Non-Coding RNAs: Multi-Tasking Molecules in the Cell

**DOI:** 10.3390/ijms140816010

**Published:** 2013-07-31

**Authors:** Anita Quintal Gomes, Sofia Nolasco, Helena Soares

**Affiliations:** 1 Health Technology College of Lisbon—Polytechnic Institute of Lisbon, 1990-096 Lisbon, Portugal; E-Mails: anita.gomes@estesl.ipl.pt (A.Q.G.); sofianolasco@fmv.utl.pt (S.N.); 2 Institute of Molecular Medicine, Faculty of Medicine, University of Lisbon, 1649-028 Lisbon, Portugal; 3 Gulbenkian Science Institute, 2780-256 Oeiras, Portugal; 4 Interdisciplinary Centre of Research in Animal Health (CIISA), Faculty of Veterinary Medicine, 1300-666 Lisbon, Portugal; 5 Center for Chemistry and Biochemistry, Department of Chemistry and Biochemistry, Faculty of Sciences, University of Lisbon, 1749-016 Lisbon, Portugal

**Keywords:** sncRNAs, lncRNAs, miRNAs, siRNAs, piRNAs, gene expression regulation, epigenetic regulation

## Abstract

In the last years it has become increasingly clear that the mammalian transcriptome is highly complex and includes a large number of small non-coding RNAs (sncRNAs) and long noncoding RNAs (lncRNAs). Here we review the biogenesis pathways of the three classes of sncRNAs, namely short interfering RNAs (siRNAs), microRNAs (miRNAs) and PIWI-interacting RNAs (piRNAs). These ncRNAs have been extensively studied and are involved in pathways leading to specific gene silencing and the protection of genomes against virus and transposons, for example. Also, lncRNAs have emerged as pivotal molecules for the transcriptional and post-transcriptional regulation of gene expression which is supported by their tissue-specific expression patterns, subcellular distribution, and developmental regulation. Therefore, we also focus our attention on their role in differentiation and development. SncRNAs and lncRNAs play critical roles in defining DNA methylation patterns, as well as chromatin remodeling thus having a substantial effect in epigenetics. The identification of some overlaps in their biogenesis pathways and functional roles raises the hypothesis that these molecules play concerted functions *in vivo*, creating complex regulatory networks where cooperation with regulatory proteins is necessary. We also highlighted the implications of biogenesis and gene expression deregulation of sncRNAs and lncRNAs in human diseases like cancer.

## 1. Introduction

### 1.1. The Incredible RNA Molecules

RNA has been known since the late 1800s, but its importance in cell functioning has long been in the shadow of DNA and proteins. In the 1950s, with the establishment of the molecular structure of DNA, it was proposed that RNA would be an intermediate molecule in the information flux between DNA and proteins. Later, this was experimentally demonstrated revealing that during gene expression, DNA is copied in a molecule of messenger RNA (mRNA) that is then translated into proteins with the help of other RNA molecules like transfer RNA (tRNA) and ribosomal RNAs (rRNAs). The idea that RNAs are much more than molecules involved in storage/transfer of information emerged with the discovery of ribozymes, RNA molecules that have, like proteins, active roles as catalysts of chemical reactions in cells. The two ribozymes identified first have RNAs as substrates and were the *Tetrahymena* intron of the 26S rRNA that is a self-sufficient catalytic unit capable of autoexcision and autocyclization [1], and the ribonucleoprotein, RNase P, an enzyme containing an RNA subunit essential for the catalysis required for the synthesis of tRNAs [2]. These discoveries clearly encouraged a variety of studies to search for potential new roles of RNA molecules *in vivo*, and led to the re-evaluation of RNAs as crucial molecules in the evolution of life. In view of the ability of RNAs to catalyze biological reactions, it is conceivable that the first organisms could rely only on RNA molecules and that only later an evolution of a more complex system based on proteins was established. This hypothesis gave support to the model of a primordial “RNA World” (for review [3,4]).

Progressively, the participation of RNAs in other critical molecular processes in eukaryotic cells was revealed, as in the case of DNA replication (RNA primers allow DNA polymerases to start the process), protein translation and RNA transcript maturation. For example, several ribosome functions required for protein synthesis were shown to be, at least in part, RNA-mediated, including peptidyl transferase activity [5], decoding functions [6], and the tRNA acceptor site interaction with 23S rRNA [7]. On the other hand, many small non-coding RNA molecules were isolated and characterized as being associated with proteins originating from ribonucleoprotein complexes (RNP), later identified as the components of the splicesome, including U1, U2, U4, U5 and U6 small nuclear RNA (snRNA) [8].

Furthermore, the information content of tRNA, rRNA and mRNA molecules can be biochemically altered after transcription by different molecular mechanisms that are generally designated by RNA editing [9]. These include sequence changes such as nucleoside modifications from C to U and A to I deaminations, as well as non-templated nucleotide additions and insertions. In general, RNA editing mechanisms are based on protein or protein-RNA complexes responsible for the RNA editing reaction and require a “guide RNA” molecule, which, through base-pairing with the target RNA molecule, determines the editing site. By this mechanism an mRNA sequence may be post-transcriptionally altered and consequently the amino acid sequence of the protein will then differ from that predicted by the genomic DNA sequence. Moreover, post-transcriptional processing and modifications of rRNAs are important for the production of efficient and accurate ribosomes which is directed by two large guide families of small nucleolar RNAs (snoRNA) [10].

In the mid-1980s Blackburn and Greider, demonstrated the existence of an enzymatic activity within cell extracts that added tandem hexanucleotides to chromosome ends and led to the discovery of telomerase [11]. Today it is well established that telomerase is a specialized reverse transcriptase that uses an internal RNA template sequence that is responsible for the synthesis of telomeric repeats [12]. More recently, Quiao and Cech [13] have described that the non-template RNA part of telomerase works together with the protein reverse-transcriptase motifs to facilitate catalysis, using a mechanism resembling that of pure ribozymes [13]. According to the “RNA first” model it was speculated that telomerase arose by the association of an ancient ribozyme with the reverse-transcriptase subunit. In light of this hypothesis the telomerase RNA may be a molecular fossil and telomerase a missing link in the evolution from RNA enzymes to protein enzymes [13]. This type of close functional collaboration is also observable in snoRNPs.

At this point the growing descriptions of the importance of RNA molecules for cell function started to push them to the limelight, but the complexity of their roles and the wide variety of molecular mechanisms where RNA molecules are critical players was still far from clear. In recent years, the use of genome wide approaches and the large output of genome sequencing technologies have revealed that the mammalian transcriptome is much more complex than previously thought since it includes a large number of small non-coding RNAs (sncRNAs) and long noncoding RNAs (lncRNAs) [14,15]. In most cases, these molecules present complex and precise patterns of expression during differentiation and development, tissue specificity, and some have been related to different pathophysiological states [16]. For example, it became clear that snoRNA guide families are widely diverse, which seems to be related to variant snoRNA structures and multiple cellular RNA targets, and consequently to cellular functions beyond ribosome biogenesis [10,17]. Indeed, snoRNAs have been recently implicated in alternative splicing and in cell transformation, tumorigenesis, and metastasis (for review, see [18]) showing that we are far from having a complete picture of their roles *in vivo*. Importantly, the observations that exogenously introduced double stranded RNA (dsRNA) molecules and plasmids expressing short hair-pin RNA (shRNA) specifically base-pairing with target mRNA molecules were able to trigger mRNA degradation (RNA interference -RNAi) [19,20] revealed, for the first time, that specific silencing pathways based on sncRNAs operate in eukaryotic cells. Moreover, these observations led to the development of the powerful RNA interference (RNAi) technique that has been extensively used in the study of gene function.

The aim of this review is to give a summarized overview of the biogenesis pathways of distinct classes of sncRNAs, including miRNA, piRNA, and siRNA, as well as lncRNAs, focusing on the miRNA and lncRNAs gene regulatory roles in distinct cellular functions and developmental regulatory programs. We will highlight the implications of the deregulation of miRNA and lncRNAs biogenesis pathways further illustrating the role of these molecules in the establishment of human diseases such as cancer. Finally, we will bring to discussion the fact that the pathways where distinct family members of sncRNAs and lncRNA funtion are probably interconnected, establishing a complex network of interactions and actions required for rapid and fine-tuned gene expression regulation at multiple levels.

### 1.2. The Small Non-Coding RNAs

Three classes of sncRNAs, namely short interfering RNAs (siRNAs), microRNAs (miRNAs) and PIWI-interacting RNAs (piRNAs), have been extensively studied in the last decade and have been associated with pathways that lead to silencing of specific genes and to the protection of the cell/genome against viruses, mobile repetitive DNA sequences, retro-elements and transposons [16].

#### 1.2.1. siRNAs and miRNAs

The siRNAs and miRNAs (~20–30 nucleotides long) originate from double-stranded RNA (dsRNA) precursors that are introduced into, or produced endogenously by gene transcription of both sense and anti-sense DNA strands and of pseudogenes and inverted repeats. These molecules are critical in pathways involved in mRNA degradation, translational repression, or both, therefore regulating gene expression.

In the case of siRNAs, they are small RNA duplex molecules produced by the action of Dicer, a ribonuclease III (RNaseIII) enzyme that creates RNA duplexes with 2-nt overhangs at their 3′ ends and phosphate groups at their 5′ ends [21].

The miRNAs are mostly transcribed by RNA polymerase II as primary-miRNA (pri-miRNA) molecule precursors that possess a characteristic stem loop structure and are subsequently subjected to processing mechanisms [22]. In animals, the first step occurs in the nucleus where the RNaseIII Drosha acts over pri-mRNAs generating a pre-miRNA, a small RNA duplex of ~65–70 nucleotides containing the hair pin. This action can be facilitated by RNA processing proteins such as hnRNP A1 [23]. The pre-miRNAs are then exported to the cytoplasm by a nuclear transport receptor complex, exportin-5–RanGTP [24] where they are processed by Dicer into ~22-nt mature miRNAs (miRNA–miRNA* duplexes, where miRNA is the antisense, or guide/mature strand, and miRNA* is the sense, or passenger strand).

An alternative nuclear pathway for miRNA biogenesis was described in invertebrates [25] where the pre-miRNA is processed via splicing/spliceosome, instead of Drosha. Accordingly, spliced lariats linearized by the lariat debranching enzyme accept monophosphates and 3′ hydroxyls, the same moieties found in pre-miRNAs, that were designated by-miRNAs/introns, “mirtrons” (for review [24]). These mirtrons are subsequently exported to the cytoplasm and processed by a Dicer protein.

The next step, for both siRNA and miRNA production, is the subsequent association with members of the Argonaute protein family that have diverged into specialized clades (or subfamilies), each recognizing different sncRNA types and conferring the specific features of the various silencing pathways operating in cells [26]. Argonaute loading occurs in the RNA-induced silencing complex (RISC)-loading complex, a ternary complex that consists of an Argonaute protein, Dicer and a dsRNA-binding protein (known as TRBP in humans). During loading, the non-guide strand is cleaved by an Argonaute protein [22].

The selection of the different Argonaute proteins seems to be based on the small interfering RNA duplex structure. For example, siRNAs that are perfect duplexes in terms of base pairing are loaded into Argonaute 2 (Ago2), whereas duplexes presenting mismatches, as in the case of miRNAs, are generally driven to Argonaute 1 (Ago1) [27,28]. When the complementarity between the miRNA bound to Ago1 and the target RNA is high, this causes miRNA tailing and 3′- to 5′-trimming. The discrimination between Ago1 and Ago2 seems to depend on the action of Hen1 an enzyme that adds the 2′-*O*-methyl group at the 3′ ends of small RNAs bound to Ago2, but not those bound to Ago1 [29]. This methyl group is known to block tailing and trimming of the miRNA. The maturation and function of certain miRNAs can be also associated to enzymatic post-transcriptional modifications, like mono-uridylation [30]. These modifications will increase the variety of miRNAs and their precursor pools allowing more complex schemes of regulation in different backgrounds.

In the small RNA duplex of the siRNA the guide strand seems to be the one whose 5′ end is less tightly paired to its complement [31]. In both siRNAs and miRNAs the guide strands drives the RISCs to the target mRNAs that contain complementary sequences thereby causing their degradation or translation inhibition (for review [16,32]). Recently, it has been shown that the target choice can also depend on accessory factors that interact with Dicer. For example, the *Drosophila* Loqs-PB Dicer-partner cleaves pre-miR-307a, generating a longer miRNA isoform with a distinct seed sequence and target specificity [33]. The mammalian TRBP homologue also acts together with Dicer to cleave pre-miR-132 generating a longer miRNA and consequently targets different mRNA molecules [34].

In fission yeast a specialized nuclear complex, known as the RNA-induced transcriptional silencing complex (RITS), mediates transcriptional gene silencing by inducing heterochromatin formation [35]. The RITS complex consists of Chp1 (H3K9me binding protein), Ago1, a poorly characterized protein Tas3, and siRNAs derived from centromeric repeat sequences [36]. These studies also showed the existence of a tight coupling of both siRNA and H3K9 methylation that appears to be important for the recruitment of RITS for heterochromatin assembly [37]. Therefore, there seems to exist a complex interplay between the RNAi pathway and the chromatin modifying machinery [37].

#### 1.2.2. The piRNAs

The piRNAs are the least characterized class of sncRNAs and, contrary to the siRNAs and miRNAs that are widely expressed in different tissues and cell types, the piRNAs have been essentially detected in the germline cells of mammals, fish and *Drosophila melanogaster* [38,39] where they are important for germ line development and to suppress transposon activity. Mutations that disrupt the piRNA biogenesis pathway in mouse and fish cause germline-specific cell death and sterility, and are also associated with increased transposon expression [40].

The piRNAs (~24–31 nucleotides) got their name from the fact that they only associate to the PIWI subfamily of the Argonaute protein family (Piwi proteins). These sncRNAs usually have a uridine at the 5′ end, hold a 5′ monophosphate, and present a 2′-*O*-methyl (2′-*O*-Me) modification on the nucleotide at the 3′ end (for review, see [32]).

Although not much is known concerning the intervening factors involved in piRNAs biogenesis pathways and transcription regulation, it is now well documented that they diverge from siRNAs and miRNAs by being generated by RNaseIII-independent pathways that do not involve dsRNA precursors. These sncRNAs are generated from long single-stranded precursors [41,42] that are preferentially cleaved at U residues and loaded onto Piwi proteins.

In *Drosophila*, as in mammals, the majority of piRNAs are transcribed from discrete genomic *loci* that are clustered in large pericentromeric or subtelomeric domains, generally spanning from 50–100 kb, and that comprise mainly various transposable DNA elements and their remnants [41]. Other piRNAs are derived from 3′ UTRs of protein coding genes and dispersed euchromatin copies of transposable elements [41,43]. Most of these clusters are active specifically in germ cells, while only a single major cluster (*flamenco*) impels transposon silencing in the soma. Interestingly, if new transposons are introduced into piRNA clusters, and if they are heritable by the progeny, novel piRNAs will be produced that can lead to the control of the new transposons indicating that the mechanisms that drive adaptation to transposon invasion might be mediated by the piRNA pathway [44].

The critical role of piRNAs on transposon silencing was demonstrated by loss-of function mutations in *Drosophila* piRNAs and genes coding for the proteins involved in their biogenesis. In the germline these mutations cause a retro-transposition up-regulation causing the loss of germ cells and a variety of defects due to alterations in microtubule cytoskeleton polarization, with consequences to the polarized localization of specific proteins and mRNAs required for normal oogenesis [45]. However, it was found that the derepression of transposons activates the Chk2 DNA damage checkpoint [44] suggesting that the described phenotypes are probably an indirect consequence of transposon overexpression and DNA damage signaling (for review [44]).

Other studies also reveal that besides being involved in keeping genome integrity, a subset of piRNA genes have been implicated in the assembly of the telomere protection complex [46].

Detailed analysis of the small RNAs associated with the Piwi sub-family (PIWI, Aubergine and Argonaute 3) [41,47] in the *Drosophila* female germline showed that these sncRNAs have in their structure and sequences, signatures that give clues about their biogenesis. The most abundant piRNAs are mainly generated from the antisense strand of retro-transposons and these preferentially associate with Piwi and Aubergine proteins [41,47]. Those present in the single major somatic cluster are mainly originated from the sense strand and are associated with Argonaute 3 (Ago3).

The piRNAs from the germ cells seem to be generated by a self-amplifying loop designated by ping-pong cycle. Specifically, PIWI and/or Aubergine form complexes with antisense piRNAs that direct the slicing of sense strand transposon transcripts [41,47]. The sliced sense strands are then bound by Ago3, and this complex directs the slicing of antisense transposon transcripts [41]. A similar mechanism seems to operate in other animal genomes [42,48]. The piRNAs derived from genomic regions depleted of transposons, seem to be generated by a different pathway not completely understood called “primary processing” that operates in somatic cells, and may have a role in the regulation of target mRNAs (for review [32]).

Recent studies have shown that, in addition to their role in germ line transposon regulation and genome stability, piRNAs have a broader function in heterochromatin formation and developmental gene regulation. The analysis of a high-throughput small RNA sequencing data in *Drosophila*, mouse and rhesus macaque samples demonstrated that piRNAs are widespread and are abundant in other tissues as much as in the germline [49]. In fact, their involvement in the regulation of gene expression was demonstrated in *Drosophila*, where the degradation of a subset of maternal RNAs, *i.e.*, embryonic posterior morphogen Nanos (Nos), at the maternal-to-zygotic transition, was shown to require the zygotic expression of a piRNA cluster [50]. When this expression is inhibited, the Nos mRNA is stabilized which was accompanied by a reduced deadenylation and translational derepression, resulting in head development defects. Because the piRNAs involved in this regulation are produced from transposable elements, the authors suggested the existence of a direct developmental function for transposable elements in the regulation of gene expression through piRNAs [50].

The importance of the piRNAs pathway in the nervous system and in epigenetic regulation has also been gaining support. In the hippocampus, the inhibition of piRNAs causes a decrease of the dendrite spine area suggesting that these sncRNAs are required for spine morphogenesis [51]. More recently, in Aplysia sensory neurons, a Piwi/piRNA complex was described to facilitate the methylation of a conserved CpG island in the promoter of the transcriptional repressor of memory, CREB2, in a serotonin-dependent manner [52]. Consequently, this Piwi/piRNA complex is at the cross-roads between a transient external stimuli and alterations in the gene-expression of neurons involved in long term memory storage. Sienski *et al.* [53] have also shown that in *Drosophila* ovarian somatic cells piRNAs mediate the silencing of hundreds of transposon copies at the transcriptional level by establishing heterochromatic methylation of H3K9 on transposons and their genomic surroundings. The involvement of the piRNA pathway in *de novo* methylation of the differentially methylated region of the imprinted mouse *Rasgrf1 locus* [54] shows that the role of this pathway in methylation is also extendable to mammalian genomes.

There is also growing evidence that piRNA-pathway dependent mechanisms may have been critical during evolution, in the establishment of developmental robustness. In fact, the piRNA-pathway seems to be required for preventing phenotypic variation despite genotypic variation and environmental influences (canalization) [55]. The Hsp90 protein was previously described as a capacitator [56] being able to prevent phenotypic variation by suppressing the mutagenic activity of transposons [57]. Interestingly, it was shown in *Drosophila* that a protein complex composed of Hsp90, Piwi and Hop, is involved in canalization, probably through phosphorylation regulation of the Piwi protein by Hsp90 and Hop [55]. Therefore, it is possible that the Piwi-piRNA pathway will mediate canalization by both suppressing the generation of new genotypes and epigenetically silencing the expression of existing genetic variants [55].

The piRNAs, contrary to miRNAs, are less conserved through the eukaryotic lineage. This difference has been explained by the possible co-evolvement of miRNA with their RNA targets which have created sequence divergence constraints. There are increasing examples that piRNAs play roles in somatic cells regulating protein encoding genes. It is possible that piRNAs are more likely to be involved in epigenetic regulation rather than post-transcriptional regulation [58]. These puzzling facts suggest that our knowledge of the mechanistic relationships between piRNAs and the regulatory mechanisms based on regulatory proteins is far from being understood. On the other hand, the initial evidence that piRNAs may be involved in epigenetic regulation in tumorigenesis [59,60] requires additional attention.

The role of piRNAs in protecting genomes against parasitic nucleic acids seems to have developed early in evolution since ciliates present a mechanism that resembles that of piRNAs. Ciliates are single celled organisms that present a polyploid macronucleus that guarantees the vegetative growth of cells (the somatic nucleus) and the diploid micronucleus that is only active during sexual conjugation and constitutes the germline [61]. After conjugation the zygotic macronucleus differentiates from the micronucleus by undergoing an extensive developmentally programmed genome reorganization [62]. This reorganization involves chromosome fragmentation and elimination of germline limited sequences (internal eliminated sequences (IES), transposons and other repeated sequences) according to the pre-existing rearrangements of the maternal somatic genome. This seems to rely on a global comparison of the germline and somatic genomes and a genomic subtraction between meiosis-specific, germline scnRNAs (small RNAs that resemble piRNAs) and longer non-coding transcripts from the somatic genome (for review, see [63]). This mechanism that parallels the patterns of heterochromatin formation in other eukaryotes allows the maintenance of an epigenetic memory of rearrangement patterns across sexual generations and establishes, in an ancestral unicellular organism, a relationship between piRNAs and development. The ancestrality of the piRNAs and the fact that they have been placed in developmental frameworks being protagonists in the establishment of developmental robustness strongly supports the view that they have been critical factors in eukaryotic evolution.

## 2. Long Noncoding RNAs

It is now clear that the mammalian genome produces a large transcriptome of long noncoding RNA (lncRNA, defined as RNA >100 nucleotides in length). The number of gene members integrating this class of ncRNAs is still under debate and ranges from 10,000 to >200,000 [64].

The lncRNAs can be transcribed from intergenic regions, promoter regions or be interleaved, overlapping or antisense to annotated protein-coding genes [44]. There is also growing evidence that lncRNAs molecules might be produced by transcriptional active pseudogenes [65]. Although the majority of lncRNAs are transcribed from the nuclear genome, recently it was found that some can be generated from mitochondrial genomes [66]. Like coding genes, lncRNAs undergo post-transcriptional processing, including 5′capping, alternative splicing, RNA editing, and polyadenylation [67,68].

The referred transcriptional origins have been used to establish classification classes for lncRNA, as for example promoter-associated long RNAs (lpaRNAs) [68], natural antisense transcripts (NATs) or opposite-strand transcripts [69], large intervening noncoding RNA (lincRNA) [70], and enhancer associated RNAs (eRNA) [71,72]. However, other criteria should probably be used since frequently one lncRNA molecule can be associated with more than one class.

Mammalian genomes encode a large number of natural antisense transcripts (NATs) [64,73]. For instance, the FANTOM-3 mouse transcriptome sequencing consortium identified natural antisense transcripts for more than 70% of the transcription units, the majority of which represent non-protein-coding RNAs [73].

NATs have been defined as endogenous RNA molecules at least partially complementary to transcripts of known function [74]. NATs can be transcribed from the opposite strand at the same genomic locus of their sense counterparts and will present perfect sequence complementarity being designated by *cis*-NATs. On the other hand those transcribed from different genomic loci may have imperfect sequence complementarity and are named *trans*-NATs [75]. Sense and antisense RNA pairs can present different relative orientations and variable overlapping regions. For example, they can overlap by their 5′ regions (5′ to 5′), by their 3′ regions (3′ to 3′), or fully-overlap (one gene included within the region of the other) [76]. Antisense RNAs have a tendency to have lost introns and typically show lower abundance compared with sense transcripts [77].

Studies performed in various organisms have suggested that NATs can participate in a broad range of regulatory events that will be discussed later.

## 3. The Emerging Roles of lncRNAS and miRNAs

### 3.1. LncRNAs: Implications in Different Levels of Gene Expression Regulation and Differentiation

LncRNAs have emerged as pivotal molecules for the regulation of gene expression [76]. These transcripts are biologically relevant as supported by their cell-specific expression pattern [78], subcellular distribution [79], developmental regulation and possible association with human diseases.

LncRNAs encompass a wide variety of functions which include almost all levels of gene expression regulation, ranging from epigenetic to translational regulation, including transcriptional and post-transcriptional control. The main functions of lncRNAs are summarized below.

#### 3.1.1. Epigenetic Regulation

lncRNAs modulate chromatin through the specific recruitment of histone and chromatin modifying complexes on one hand and by the recruitment of transcription factors on the other hand. X chromosome inactivation (XCI) is the classic example of the former type of regulation and is caused by the lncRNA “Xist” which physically associates with the Polycomb repressive complex 2 (PRC2) recruiting it to the X chromosome ultimately leading to its inactivation [80]. More precisely, it is a 1.6-kb ncRNA (RepA) within Xist that targets PRC2. Depletion of RepA abolishes full-length Xist induction and trimethylation on lysine 27 of histone H3 of the X (thus abolishing X inactivation). In addition it was demonstrated that PRC2 deficiency compromises Xist up-regulation [80]. A similar process to XCI is genomic imprinting, an epigenetic event in which genes are expressed from the allele of only one parent. One of the first lncRNAs to be identified was H19, which is reciprocally imprinted with insulin-like growth factor 2 (Igf2). Even though this lncRNA is highly expressed, its deletion has no phenotype and, in fact, recently it has been proposed to function as a microRNA precursor [81].

Other lncRNAs (*i.e.*, Air, Kcnq1ot1, HOTAIR) can control chromatin states in *cis* and/or in *trans*, thereby regulating gene expression through the association with chromatin-modifying complexes [82,83]. Specifically, HOTAIR is a *trans-*acting lncRNA that serves as a scaffold for two histone modification complexes: it binds both to polycomb repressive complex 2 (PRC2) and to LSD1 (in complex with CoREST/REST). This coordinates targeting of PCR2 and LSD1 to chromatin for coupled histone H3 at lysine 27 methylation and lysine 4 demethylation leading to subsequent gene silencing [84]. Also, in the plant *Arabidopsis* it was demonstrated that environmental conditions, such as cold, are able to induce the transcription of related NATs (*i.e.*, COOLAIR) that are involved in the silencing of a flower repressor *locus* designated by flowering locus c (*FLC*) [85]. More recently it was discovered that a lncRNA, named COLDAIR, that differs from COOLAIR by the fact that it is transcribed in the sense direction relative to *FLC* mRNA transcription, interacts directly with PRC2 and targets it to *FLC*, establishing an epigenetic memory [86]. Interestingly, winter cold triggers the methylation of H3 at *FLC* and it was shown that COLDAIR is induced by cold, demonstrating that lncRNas participate in the integration of signals from the environment to cell signaling pathways.

Other *trans-*acting lncRNAs have different functions some of which remain incompletely defined. For example, the p21-associated ncRNA DNA damage-activated (PANDA) lncRNA is induced upon DNA damage in a p53-dependent manner and it interacts with the transcription factor NF-YA to limit expression of pro-apoptotic genes [87]. Mistral is another example of an lncRNA that acts on the recruitment of the transcription factor MLL1 thereby activating Hoxa6 and Hoxa7 expression and subsequent stem cell differentiation [88].

Another group of lncRNAs that play a role in mammalian genomes are the long intergenic non-coding RNAs (lincRNAs) that range in size from ~300 nucleotides to several thousands and that, in humans, have been estimated to be around 3300, although a more correct number may be closer to 4500 [89]. This group of transcripts is heterogeneous but show significant evolutionary conservation relative to neutral sequences [70], which support the idea that they have important functions. In fact, it has been described that some groups of lincRNAs present expression patterns that correlate with those observed for protein-coding genes involved in cellular processes as diverse as cell-cycle regulation, innate immunity responses, and stem cell pluripotency [70,90]. In agreement, a reference catalog of 8195 human lincRNAs based on integratingRNA-seq data from 24 tissues and cell types showed that lincRNAs are expressed in a more tissue-specific manner than protein-coding genes [91]. By using co-immunoprecipitation and RNAi approaches it was also demonstrated that lincRNAs are associated with chromatin-modifying complexes to specific genomic loci to regulate gene expression [89]. The capacity to bind chromatin-modifying proteins or transcription factors, as exemplified, in combination with the abundance of lncRNAs suggests that lncRNAs may be part of a broad epigenetic regulatory network (reviewed in [92,93]).

#### 3.1.2. Transcriptional Regulation

The discovery and characterization of several ncRNAs that are able to associate with promoters (promoter associated RNAs—paRNA) is also changing the traditional view of how genes encoding proteins are regulated at the transcriptional level. Promoter associated RNAs paRNAs are transcribed approximately from the start of or within the promoter, and include long, short and tiny RNA molecules (for review [49]). The long paRNAs were found at a single-gene level and were also associated with the modification of DNA methylation and demethylation patterns [94], inhibition of transposition expression in *Saccharomyces cerevisiae* [95] and gene expression in humans [96].

Interestingly, long (antisense) paRNAs have the potential to form double stranded molecules that can be processed into endo-siRNAs, and that, due to their sequence complementarity to that of a promoter, are able to induce transcriptional gene silencing [97–99] or activation [100–102] in a similar way to short paRNAs [49]. This picture is far from being complete since an increasing amount of experimental data are supporting the idea that enhancers can be transcribed and the resulting enhancer-non coding transcripts (eRNAs) may, in some cases, have functional roles, rather than represent mere transcriptional noise (for review see [103,104]).

On the other hand, lncRNAs can modulate the function of transcription factors by acting as co-regulators, modulators of transcription factors activity or by regulating the association and activity of co-regulators, among others. The ncRNA Evf-2, for example, functions as a co-activator for the homeobox transcription factor Dlx2, which plays important roles in forebrain development and neurogenesis [105]. Local ncRNAs can also recruit transcriptional factors and co-activating molecules to regulate adjacent protein-coding gene expression. The RNA binding protein TLS, binds to and inhibits the CREB binding protein (CCND1) and p300 histone acetyltransferase activities on a repressed gene target, cyclin D1. The recruitment of TLS to the promoter of cyclin D1 is directed by single stranded, low copy number lncRNA transcripts tethered to 5′ regulatory regions of CCND1 in response to DNA damage signals [106].

Finally, lncRNAs also regulate the basal transcription machinery by targeting transcription factors required for the RNAP II transcription of all genes [107]. These general factors include components of the initiation complex that assemble on promoters or are involved in transcription elongation. An example of lncRNA-mediated regulation of basal transcription is the formation of a stable RNA-DNA triplex within the major promoter of the dihydrofolate reductase (DHFR) by an lncRNA that is transcribed from an upstream minor promoter of the DHFR gene. This complex prevents the binding of the transcriptional co-factor TFIIB [96].

#### 3.1.3. Post-Transcriptional Regulation

LncRNAs can act on splicing, on mRNA stability and translation. It has been shown that lnc antisense RNA may bind to the sense RNA, masking the splice sites and thereby changing the balances between splice variants. Thyroid hormone receptor alpha gene (*TRα*) is an example where the antisense transcript *RevErbAα* influences splicing of *TRα1* and *TRα2* mRNAs [108]. Recently, it was discovered that a new class of sno-lncRNAs, whose ends correspond to positions of intronic snoRNA, are able to interact with the splicing factor Fox2 and alter splicing patterns [109]. The authors also showed that some of these sno-lncRNAs map to a genomic region that is deleted in the patients presenting Prader-Willi syndrome, strongly suggesting an association of these sno-lncRNAs with the disease. LncRNAs can also recruit proteins to mRNA to promote its degradation or stabilization. There’s evidence for lncRNA binding to sequences present in the 3′ UTR of specific mRNAs, thus creating a recognition site for Staufen, a protein that binds double-stranded mRNA and induces its decay [110]. By contrast, the lncRNA TINCR (terminal differentiation-induced ncRNA) also interacts with Staufen 1 but the complex between TINCR-STAU1 seems to mediate stabilization of mRNAs encoding differentiation factors such as Keratin 80 [111]. TINCR-mRNA interaction occurs through a motif of 25 nt that is abundantly present in target interacting mRNAs [111]. Another example is that of the mRNA of BACE1, a β-secretase responsible for β-amyloid production, that is stabilized and protected from RNase cleavage by base pairing of its antisense (BACE1-AS) [112]. Therefore, different lncRNAs are able to differentially regulate factors involved in mRNA stability regulation. Translational regulation is yet another proposed function for lncRNAs. Such is the case of the antisense for *PU.1* mRNA. *PU.1* mRNA translation is inhibited by a noncoding antisense transcript, which is a polyadenylated RNA with a lower concentration but a half-life longer than the sense *PU.1* transcript [113]. On the other hand the lncRNA Uchl1, shuttles from the nucleus to the cytoplasm under the control of the mTOR pathway and is involved in the translation up-regulation of the ubiquitin carboxy terminal hydrolase L1 (UCHL1) mRNA by promoting its association with polysomes [114]. Interestingly, the UCHL1 is a specific neuronal protein involved in rampamycin neuroprotective function and more generally in cellular stress response, that has been associated with neurodegenerative diseases. The various referred examples clearly show that lncRNAs present a vast repertoire of strategies to post-transcriptionally regulate protein encoding genes and different molecules are able to differentially modulate a specific regulatory molecule or pathway.

#### 3.1.4. Modulation of mRNA Nuclear Trafficking and Control of Nuclear Compartmentalization

NRON is a non-coding repressor of nuclear factor of the activated T cells (NFAT), which interacts with multiple proteins including members of the importin-beta superfamily and likely functions as a specific regulator of NFAT nuclear trafficking [115].

The lncRNA nuclear-enriched autosomal transcript 1 (NEAT1), and abundant 4 kb ncRNA, is retained in nuclei *foci* that are coincident with “paraspeckles” [79]. It has been demonstrated that it contributes to the formation of these dynamic structures of the interchromatin space that are implicated in mRNA retention [79].

#### 3.1.5. Formation of Endogenous siRNA

**Figure 1:**
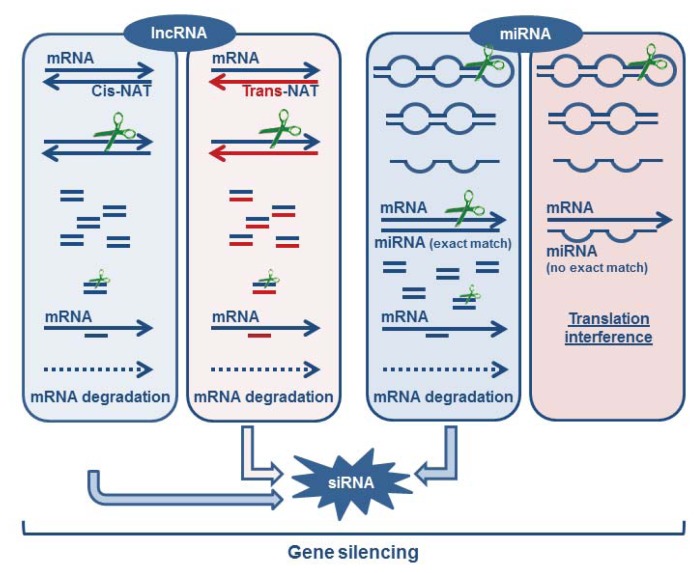
Gene silencing: mRNA post-transcriptional regulation by lncRNA and miRNA. lncRNAs can be transcribed as natural antisense transcripts, from the same loci (*cis*-NAT, the same gene is transcribed in both directions) or from a different loci (*trans*-NAT, for example from a pseudogene). These NATs transcripts can pair with the coding transcripts, originating dsRNA molecules that will activate the siRNA machinery leading to mRNA degradation. miRNAs are also complementary of coding mRNAs and can pair with a perfect match leading to the activation of the siRNA machinery or they can pair with gaps leading to translation interference.

It has been described that NATs can originate siRNAs that will be involved in mRNA down-regulation. This mechanism requires the formation of a sense:anti-sense pair of transcripts that are then processed into siRNAs. This pair can be originated directly from the same *loci* (*cis*-NATs) or from different *loci* (*trans*-NATs). Interestingly, it was observed that certain *trans*-NATs are produced from pseudogene transcription. For example, in rice, a small number of pseudogenes are transcribed and processed into siRNAs, after pairing with the coding gene or a paralogous pseudogene transcript [116]. A similar observation was reported in mammals where pseudogene transcripts can be processed into small interfering RNAs (siRNA) with the ability to repress gene expression in mouse oocytes [117,118]. Therefore, NATs play their gene expression regulatory role through a mechanism equivalent to that of miRNAs and siRNAs (see Figure 1). Until recently, pseudogenes were envisaged only as copies of protein-coding genes that have lost the ability to produce functional proteins therefore constituting junk DNA in genomes [119]. Pseudogenes can be created by diverse processes, including: (1) spontaneous mutations, preventing transcription of the gene, or translation of the protein [120]; (2) duplication, in which pseudogenes are originated via tandem duplication or uneven crossing-over leading to the loss of promoters or enhancers or the appearance of crippling mutations such as frame shifts or premature stop codons [119]; and (3) retro-transposition, the mRNA transcript being reverse-transcribed and integrated into the genome at a new location originating retro-transposed or processed pseudogenes [121,122]. Therefore, their origin directly makes them prone to participate in post-transcriptional regulatory mechanisms promoted by lncRNAs. These observations started to change the vision that pseudogenes are mere junk in the genomes of organisms, and suggested that they can play important biological roles. In agreement with this hypothesis is the fact that the transcription of NATs is generally regulated in a tissue-specific manner and varying sense/antisense ratios are found [123].

It is evident that the critical roles of lncRNAs at different levels of gene expression regulation will largely contribute to establish differential profiles of gene expression required for development [124–127]. This is supported by the observation that lncRNAs such as Xist [128], TUG1 [129], PINC [130], and HOTAIR [131] have important roles in development. Moreover, Dinger *et al.* [90] using a microarray to examine the expression profiles of mouse embryonic stem cells differentiating as embryoid bodies over a 16 day time course have identified 945 ncRNAs, of which 174 were differentially expressed, many correlating with pluripotency or specific differentiation events [90]. Accordingly, it was also observed that the expression of some lincRNAs is increased in induced pluripotent stem cells (iPSCs) in comparison to those found in stem cells. This suggests that their activation may promote the emergence of iPSCs. It was also demonstrated that one of this lincRNA (lincRNARoR) modulates the reprogramming process leading to pluripotent stem cells [132].

Spermatogenesis is a very complex developmental process that requires precise microtubule cytoskeleton remodeling, creating complex microtubule structures such as the manchette and the flagellum of the sperm [133]. During this process it was observed that the gene encoding TBCA, a protein that interacts with β-tubulin and is involved in the folding and dimerization of new tubulin heterodimers (the building blocks of microtubules) is regulated by a *Tbca* pseudogene that is transcribed in both directions [134]. The *Tbca* pseudogene is down-regulated leading to the increase of the *Tbca* mRNA, during testis maturation suggesting that this *Tbca* lncRNA is required for the undifferentiated state of spermatids. Similarly, the gene encoding the nitric oxide synthase protein (NOS2A) is transcribed into a noncoding RNA containing a region of significant antisense homology with the NOS2A mRNA. As in the case of *Tbca* lncRNA, the expression patterns of the anti-NOS2A RNA and the NOS2A mRNA exhibit opposite changes in undifferentiated human embryonic stem cells (hESCs) and in hESCs induced to differentiate into neurogenic precursors [74]. In conclusion, lncRNAs are clearly required to regulate programs of differentiation during development and seem to be generally associated with the undifferentiated states, repressing critical target genes whose expression is crucial for the cells to reach their fate.

### 3.2. miRNAs as Critical Regulators of Target Degradation and Translation

miRNAs act as sequence-specificity guides for the RNAi machinery to mediate repression of target gene expression. First identified as regulators of larval development in nematodes [19], miRNAs are now known to serve key roles in the regulation of almost every important cellular process in all multicellular eukaryotes. These include cell development, proliferation, differentiation, apoptosis and oncogenic transformation [135]. The genome of human cells encodes over 1000 miRNA species that regulate 60% of all protein-coding genes [32]. Most mRNA targets contain multiple miRNA binding sites, and each miRNA can regulate multiple genes. Therefore, the deregulation of miRNA levels might perturb the expression of many genes, thereby playing a key role in the occurrence of diseases (see below).

It is still unclear whether miRNAs act mainly at the mRNA translational or transcriptional levels. The miRNA repression, at the level of transcriptional inhibition, can occur as a consequence of mRNA decay, direct mRNA cleavage or through miRNA-mediated chromatin reorganization. Decay of targeted mRNA occurs without direct cleavage at the binding site. Unlike in translational inhibition where only a slight protein decrease can be obtained, protein level reductions greater than 33% indicate that mRNA decay is the major component of miRNA-driven silencing [136]. miRNA-mediated mRNA decay can occur via deadenylation, decapping or 5′ to 3′ degradation of the mRNA [137]. Dicer1, Ago1 and Ago2 were shown to be required for the rapid decay of mRNA containing AU-rich elements (AREs) in the 3′ UTR of tumor necrosis factor-alpha suggesting that miRNA targeting of ARE is essential to mediate mRNA degradation [137]. It was also shown that upon GW182 interaction with AGO1, there is recruitment of deadenylases and decapping enzymes, leading to mRNA degradation [138]. The mRNA cleavage, another miRNA transcription repressive mechanism that is rare in animals, but frequent in plants, normally occurs when there is full complementarity between the miRNA and its mRNA target [139]. miRNAs also have the capacity to reorganize chromatin by increasing methylation of the targeted mRNA promoters thereby inhibiting their expression [140].

Finally, the repressed mRNAs, Ago proteins and miRNAs are frequently accumulated in processing bodies (P-bodies), which are cytoplasmic structures enriched in the mRNA degradation machinery but where the translational machinery is normally absent [141].

The second major mechanism of miRNAs activity includes repression of translation initiation and/or elongation, premature termination and nascent polypeptide degradation. Inhibition of translation initiation can occur at the level of cap-40S association or via 40S-AUG-60S association. Endogenous let-7 micro-ribonucleoproteins (miRNPs) or the tethering of Ago proteins to reporter mRNAs in human cells inhibit m(7)G-cap-dependent translation initiation, suggesting that miRNPs interfere with the recognition of the cap [142]. The cap-binding protein eukaryotic initiation factor 4E has in fact been proposed as a molecular target of miRNA function [143]. Ago2 represses the initiation of mRNA translation by directly binding to the m(7)G-cap of mRNA targets, thus likely precluding the recruitment of eIF4E [144]. Another Ago1 was shown to interact with GW182, this interaction being essential for miRNA-mediated inhibition of translation [145]. It was also shown that miRNA-repressed mRNAs contain 40S but not 60S components suggesting that miRNAs repress translation initiation by preventing the 60S subunit from joining to miRNA-targeted mRNAs [146]. It has also been reported that some miRNAs can inhibit translation initiation by inducing the formation of dense miRNPs (pseudo-polysomes) [147]. The fact that various studies showed repressed mRNA targets to be associated with polyribosomes seems to indicate that miRNAs can also repress translation at the elongation step [148–150]. Silencing by miRNAs can also occur before completion of the nascent polypeptide chain causing a decrease in translational read through at a stop codon, with ribosomes on repressed mRNAs dissociating more rapidly after a block of initiation of translation, than those of control mRNAs [149]. These observations pinpoint a role for miRNAs in ribosome drop-off-mediated repression.

Intriguingly, there is also evidence for transcriptional [100] and translational [151] activation by miRNAs. The miRNA-373 was shown to induce expression of genes with complementary promoter sequences [100]. miRNA-10a can bind to the 5′ UTR of ribosomal protein mRNAs and enhances their translation [152]. Further, a growing series of studies has demonstrated that miRNAs and their associated complexes (microRNPs) elicit alternate functions that enable stimulation of gene expression in addition to their assigned repressive roles [151,153].

While the global importance of miRNAs is clearly illustrated by the developmental failure of Dicer-deficient embryonic stem cells (*in vitro*) and embryos (*in vivo*) [154], unique spatial and temporal expression patterns in distinct hematopoietic and neuronal lineages are clearly suggestive of multiple roles for miRNA in hematopoiesis, immune responses and neurological differentiation. The specific profiling of hPSCs by microarray and sequencing methods has allowed the identification of miRNAS that have potential roles in differentiation and development (reviewed in [155]). Several miRNA families, including the human (hsa)-miR-302, hsa-miR-106, hsa-miR-372, hsa-miR-17, hsa-miR-520, hsa-miR-195 and hsa-miR-200 families [155] were up-regulated specifically in hPSCs compared to mature differentiated cell types. Interestingly, the “seed” sequences (short sequence at nucleotides 2–8 on the 5′ end of the miRNA that binds to the 3′ UTRs of their target mRNAs) for most of these miRNAs are closely related, suggesting that these miRNA families may share mRNA targets. Thus, their regulatory functions might help maintain the unique characteristics of PSCs. Contrary to the miRNA families the hsa let-7 family [155] is expressed at significantly lower levels in hPSCs than in differentiated cells. The miRNA-dependent post-transcriptional gene regulation is also crucial for neural and immune cell development. Early evidence for miRNA function in the nervous system development came partly from knockout mutations of the miRNA processing genes present in the miRNA pathway. Pioneering studies of nervous system development using maternal-zygotic mutants of zebrafish dicer revealed gross morphological defects specifically in early brain patterning and morphogenesis [156]. Detailed studies of later stages in neural development have begun to suggest a more extensive contribution of miRNAs in the formation of synaptic connections, circuit maturation, and the activity-driven plasticity of these connections. For example, the mRNA processing enzyme DGCR8 mutant mice exhibited abnormalities in synaptic connectivity due to a reduction in the number and size of dendritic spines, reduced synaptic complexity, impaired synaptic transmission, and altered short-term plasticity [157].

In the immune system, miRNAs mediate the regulation of T cell development and function, as confirmed by the observation of defective thymic and peripheric T cell subsets in Dicer deficient mice [158,159].

Individual miRNAs play different roles at distinct developmental stages. For example, miR-125b and miR-132 regulate dendritic spine development. More specifically, miR-125b and miR-132 (as well as several other miRNA) are associated with fragile X mental retardation protein (FMRP) in mouse brain. The miR-125b overexpression results in longer, thinner processes of hippocampal neurons. FMRP knockdown is shown to ameliorate the effect of overexpressed miR-125b and miR-132 on spine morphology. It has been proposed that miR-125b negatively regulates its target, NR2A, along with FMRP and AGO1 [160].

Focusing on T cells, miRNA expression patterns vary among stages of development and T cell subsets, which indicate that these molecules may contribute to the identity of the cell subsets or their functional state [161]. Consistent with this, recent reports have demonstrated that various miRNAs, namely miR-101, miR-150, miR-155, miR181a, miR-29a, miR-146a and miR-326, are expressed in particular T cell subsets and regulate several aspects of their differentiation and function [162–164].

Like lncRNAs, miRNAs are required to regulate differentiation programs during development. However, they are associated with both undifferentiated and differentiated states repressing target genes involved in maintaining those programs.

## 4. ncRNAs Active Players in Cancer and Other Human Diseases

The deregulation of gene expression networks, responsible for normal cellular identity, growth and differentiation leads to cancer. The large majority of genome-wide association studies (GWAS) identify cancer risk loci outside of protein-coding regions. Of 301 single-nucleotide polymorphisms (SNPs) currently linked to cancer, only 12 (3.3%) change the protein amino-acid sequence. Most are located in the introns of protein-coding genes (40%) or intergenic regions (44%), raising the question of the function of these noncoding loci and their role in cancer development [165]. These facts, associated with the observations that miRNA and lncRNAs are involved in programs of differentiation and development soon raise the hypothesis that alterations in their profiles of expression could be correlated with cancer development. In the last years, numerous evidences have confirmed this hypothesis since miRNas and long ncRNAs, that present tissue-specific expression, were found to be deregulated in distinct types of cancers. For example, data coming from microarray expression from a wide range of distinct cancers showed that alterations in miRNAs are almost always present in the analyzed tumors [166]. More specifically, overexpression of miR-155 was reported in hematopoietic cancers, breast, lung and colon cancer [167], whereas miR-21 was found to be overexpressed in glioblastoma and to have antiapoptotic properties [168,169]. Also, transgenic mice overexpressing miR-17-92 developed lymphoproliferative disorders [170] and retroviral overexpression of the cluster accelerated lymphoma formation. The miR-17-92 cluster was also found to be overexpressed in lung, colon and gastric cancer [171]. Like miRNAs, lncRNAs have also been associated with cancer development. For example, the lncRNA MALAT1 is up-regulated in several cancer types and its overexpression has been linked to an increase in cell proliferation and migration in lung and colorectal cancer cells [165]. These phenotypes are probably related to the role of MALAT1 in controlling alternative splicing of pre-mRNAs [172]. However, this relationship is probably too simplistic since a more recent study indicates that MALAT1 may also have a role in the regulation of gene expression, different from alternative splicing, in lung metastasis [173].

Many studies have also shown that miRNA and LncRNAs themselves can function as tumor suppressor genes or oncogenes, [174–176]. Several studies found that the tumor suppressor p53 transcriptionally regulates the three gene members of the miR-34 family. On the other hand, the miR-34 activation resembles p53 activity, including induction of cell-cycle arrest and promotion of apoptosis, and loss of miR-34 can impair p53-mediated apoptosis [177]. However, the interaction between p53 and miR-34 is much more complex since mice possessing the combined loss of all three miR-34 members are viable and fertile, do not display morphological defects and are not prone to spontaneous tumor formation [178].

Similarly to miRNAs, some lincRNAs are transcriptional targets of p53 like the lincRNA-p21 that plays a role as a transcriptional repressor in the p53 pathway by triggering apoptosis. The lincRNA-p21 binds to hnRNP-K that allows for the correct localization of hnRNP-K, probably by influencing their target preference, and therefore the transcriptional repression of p53-regulated genes [179]. The precise mechanism by which lincRNA-p21 contributes to repression at specific loci remains to be defined.

Although most of the mechanisms that implicate lncRNA in cancer biology are uncovered, the growing available data show that they are probably linked for example to chromatin remodeling. For example lncRNAs that are known to be involved in the recruitment of epigenetic modifiers to specific loci such as ANRIL, XIST, HOTAIR and KCNQ1OT1 were observed to have modified expression in a variety of cancers [176]. Also the lncRNA named TERRA, which binds telomerase, inhibiting its activity *in vitro* [180] is downregulated in many cancer cells which may be related to the longevity of cancer cells.

**Figure 2:**
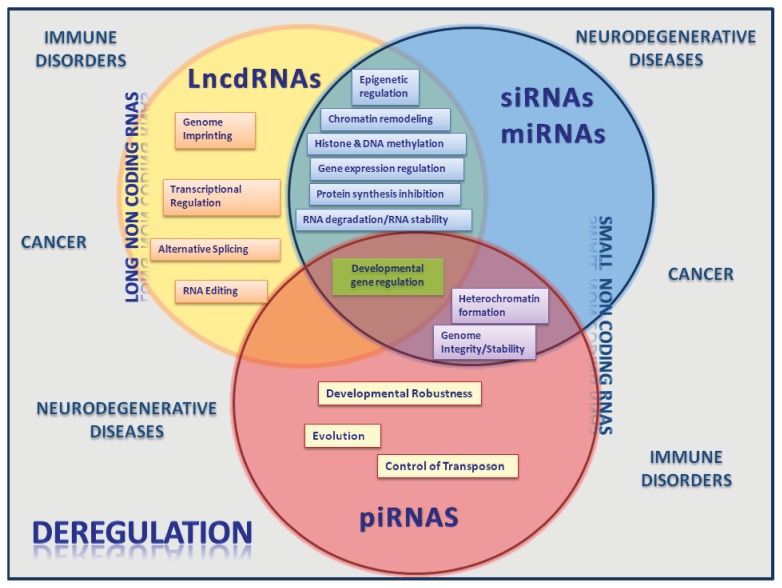
Diagram of functional relationships among lncRNAs, siRNAs, miRNas and piRNAs. This “venn diagram” depicts the specific function of each RNA molecule (inside each circle) as well as the shared functions (overlapping areas). Some of the disorders caused by deregulation in the expression patterns of these RNA molecules are indicated outside the circles.

The broad functional classes of genes and regulatory pathways that involve ncRNA participation clearly justifies that the deregulation of their biogenesis and roles could are not restricted to cancer development (Figure 2). Perturbations in the biogenesis and actions of ncRNAs have also been associated with diverse neurodegenerative diseases such as Huntington’s disease [181], Alzheimer [182] and Parkinson [183]. Moreover, recent studies have shown that miRNAs have unique expression profiles in cells of the innate and adaptive immune systems, suggesting that these molecules are important regulators of immune cell functions (reviewed in [184]). In fact, the role of miRNAs have been linked to autoimmune disorders (e.g., systemic lupus erythematosus, rheumatoid arthritis, multiple sclerosis, inflammatory bowel disease, psoriasis) and inflammatory pathologies of distinct organ (e.g., atherosclerosis, osteoarthritis, atopic eczema) and/or systemic locations like allergy. Chromatin remodeling by lncRNA is not exclusively related with cancer but is also linked to other diseases like facioscapulohumeral muscular dystrophy (FSHD) [185], lethal lung developmental disorder [186] and the HELLP syndrome, a pregnancy-associated disease [187].

The presented examples directly implicate long ncRNA and miRNAs in cancer biology and other human diseases and indicate that a complex interplay between their biogenesis pathways, their regulatory mechanisms and their targets should be seriously taken into consideration not only in cancer research but in other human pathologies and also in the definition of future strategies of diagnostics and therapeutics.

## 5. Concluding Remarks

In the last years we have witnessed an unprecedented discovery of numerous functions of non-coding RNAs in eukaryotic cells ranging from gene expression regulation to genome imprinting roles that were previously attributed to proteins.

This means that proteins are likely to cooperate with ncRNAs to control gene expression at different levels of regulation. They cooperate in the regulation of the transcription of genes encoding proteins, to process and maturate their transcripts and finally to regulate their mRNA stability and translation. Moreover, upstream of these regulatory steps, cooperation will also be required for altering DNA methylation profiles and the remodeling of chromatin contributing to epigenetic regulation. This means that complex networks between proteins and RNAs have been established during the course of evolution. We can envisage and speculate that due to their biochemical nature and biogenesis, ncRNAs will contribute to speed-up, make more flexible, transform and ultimately make more accurate the regulatory pathways conducted by regulatory proteins, pushing gene expression regulation to a new level. It is predictable that this complex regulatory web will have several hubs that will be composed of ncRNAs and proteins or alternatively only proteins or ncRNAs, which will also allow a rapid and better integration of different environmental signals. Although, the field of ncRNAs has been growing fast we are still far from understanding the complexity and the mechanisms underlying the establishment of the regulatory networks between RNAs and proteins.

From the evolutionary point of view it seems that the “invention” of proteins like telomerase was a critical step in the establishment of accurate spatial and temporal regulatory processes which probably allowed the evolution of eukaryotic complexity and later on, the appearance of multicellularity. In the view of an “RNA World hypothesis” it is tempting to speculate that the first RNA activities were to maintain the viability and integrity of “cell precursors” defending them from destructive invader molecules; these ancestral defense functions of ncRNAs that are still present and operate in “modern cells” seem to have been extended to mechanisms of gene regulation.

It should also be pointed out that the close analysis of different classes of ncRNAs (sncRNA and lncRNA), and the fact that we can detect biogenesis (see Figure 1) and functional overlaps between them (see Figure 2), strongly supports the idea that they could also have close interactions, not only at the level of their biogenesis pathways, but also at the functional level. This has been probably missed to a certain extent by the fact that they have been essentially separately studied.

From what has been compiled, the deregulation of biogenesis and functional roles of ncRNAs were, as expected at the crossroads of different human pathologies ranging from cancer to neurodegenerative and immune diseases. Finally the continued understanding of the molecular mechanisms and signaling pathways where ncRNAs participate should offer new insights to define new diagnostic strategies and open new avenues for therapies.
